# Use of healthcare resources in a cohort of rheumatoid arthritis patients treated with biological disease-modifying antirheumatic drugs or tofacitinib

**DOI:** 10.1007/s10067-020-05432-6

**Published:** 2020-09-30

**Authors:** Jorge Enrique Machado-Alba, Manuel E. Machado-Duque, Andres Gaviria-Mendoza, Juan Manuel Reyes, Natalia Castaño Gamboa

**Affiliations:** 1 Grupo de Investigación de Farmacoepidemiología y Farmacovigilancia, Universidad Tecnológica de Pereira - Audifarma SA, Pereira, Colombia 660003; 2 Grupo Biomedicina, Facultad de Medicina, Fundación Universitaria Autonoma de las Américas, Pereira, Colombia; 3 Pfizer of Colombia, Bogota, Colombia

**Keywords:** Antirheumatic agents, Biologic drugs, Disease-modifying antirheumatic drugs, Healthcare cost, Rheumatoid arthritis

## Abstract

**Introduction/objectives:**

The objective of this study is to describe the treatment patterns and use of healthcare resources in a cohort of Colombian patients with rheumatoid arthritis (RA) treated with biological disease-modifying antirheumatic drugs (bDMARDs) or tofacitinib.

**Method:**

This is a descriptive study from a retrospective cohort of patients diagnosed with RA who were treated with bDMARDs or tofacitinib after failure of conventional DMARDs (cDMARDs) or first bDMARD. Patients who were receiving pharmacological treatment between 01 January 2014 and 30 June 2018 were included. The analysis is through the revision of claim database and electronical medical records. Demographic and clinical data were collected. The costs of healthcare resources were estimated from the billing expense of healthcare service provider.

**Results:**

We evaluated 588 RA patients on treatment with bDMARDs (*n* = 505) or tofacitinib (*n* = 83), most of them were in combination with cDMARDs (85.4%). The 88.1% were females and mean age was 57.3 ± 12.5 years. The median evolution of RA since diagnosis was 9 years (IQR:4–17.2). The mean duration of use during follow-up of the bDMARDs or tofacitinib was similar, with a mean of 9.8 ± 1.9 months. It was identified that 394 (67.0%) discontinued therapy. The average annual direct cost of care per patient was USD 8997 ± 2172, where 97.2% was due to drug costs. The average annual cost of treatment per patient with bDMARDs was USD 8604 and tofacitinib was USD 6377.

**Conclusions:**

In the face of a first failure of cDMARD, bDMARDs are frequently added. A high frequency of patients do not persist treatment during the first year of follow-up. The pharmacological treatment is the most representative cause of healthcare costs.

**Table:** 

**Key Points** *• Rheumatoid arthritis is a disease with a high burden of comorbidities, complications, and worse health-related quality of life and is associated with elevated healthcare costs*. *• The biological disease-modifying antirheumatic drugs or tofacitinib medications are indicated for those with significant progression of the disease and when there is a need for alternatives to achieve low levels of activity and remission*. *• Patients with rheumatoid arthritis treated with biological disease-modifying antirheumatic drugs or tofacitinib represent a significant economic burden to the health system, especially in the costs derived from pharmacological treatment*.

## Introduction

Rheumatoid arthritis (RA) is a chronic, progressive autoimmune disease characterized by persistent synovial and systemic inflammation associated with joint destruction. It has an estimated prevalence between 0.5% and 1% in the adult population, mainly affecting women and the elderly [1, 2].

Disease-modifying antirheumatic drugs (DMARDs) are available for pharmacological treatment [3–6]. They have been classified according to their origin as biological (bDMARD) and conventional (cDMARD). The bDMARDs are used as a second line in patients who have not responded to cDMARDs, but with higher costs and incidence of adverse effects. The objective of the therapy is to control and reduce synovitis and systemic inflammation, prevent joint damage, improve quality of life, and reduce symptoms with improvement in the disability [1, 7].

Patients with RA have overexpression and overproduction of tumor necrosis factor (TNF), with interaction between B lymphocytes and T lymphocytes, and increased production of proinflammatory cytokines such as interleukin-1 and interleukin-6 [8, 9]. For these reasons, researchers designed bDMARDs, which are monoclonal antibodies that inhibit TNF, such as adalimumab, etanercept, infliximab, certolizumab, and golimumab. The inteleukin-6 inhibitors such as tocilizumab and the interleukin-1 inhibitors such as anakinra have similar effects, as do anti-CD-20 antibodies (rituximab) and cytotoxic T lymphocyte-associated antigen 4 blockers (abatacept). Also available is tofacitinib, which is classified as a targeted synthetic DMARD (tsDMARD) and oral therapy that specifically inhibits Janus kinases to block the activation of signal transduction pathways induced by cytokines [1, 5, 10–13].

In cohorts of Colombian patients, these therapies can be highly effective, leading to remission of their disease, despite adverse reactions and costs [5, 14]. Therefore, it is important to know in greater depth the factors that can influence access to and costs of healthcare for those patients who use DMARDs.

RA generates a significant economic burden for health systems, due to direct costs (healthcare and drugs) and indirect costs (productive losses), with an estimated yearly cost of USD 41,000 per patient in the United States, and this is expected to increase due to increased use of biological drugs, comorbidities, increased age of the population, and widespread physical inactivity [15, 16]. In Colombia, Montoya et al. calculated the direct healthcare costs for RA patients in Medellin between 2007 and 2009, finding a mean of USD 8178 USD per year, 87.9% of which was pharmaceutical expenditures, in particular drugs of biological origin [17]. However, there is a gap in knowledge related to the economic evidence in the current attention of RA, considering the implementation of price drug regulation, availability of treatments in the last years, and changes of attention models in the Colombian healthcare system.

Persistence is another determining factor in RA treatment, which seeks to measure the time from initiation to discontinuation of therapy [18]. This measurement is highly associated with clinical effectiveness, adverse events, patient preference, and access barriers [19]. The persistence of bDMARDs has been estimated in different observational studies between 32 and 90.9% [20]. Despite of the relevance of this variable, it has not been measured in the country.

Considering the little evidence available in these facts, the objective of this study was to determine the treatment patterns, persistence, and the use of health resources in a cohort of Colombian patients with a diagnosis of RA treated with bDMARDs or tofacitinib between 2014 and 2018.

## Materials and methods

This is an observational study which collected information from a retrospective cohort of 2200 patients who were treated in a specialized healthcare service provider (Institución Prestadora de Servicios Especializada: IPS-E) of Audifarma SA in Colombia, who were receiving pharmacological treatment for RA between January 2014 and June 2018.

The following inclusion criteria were considered: patients of either sex, older than 18 years, with a diagnosis of RA (diagnosis in IPS-E according to EULAR criteria) based on the International Classification of Diseases (ICD-10) codes M06.9, M0.690-M06.99, M05, and M06.0 and their subclassifications, who have not responded to cDMARDs or who have received only a first bDMARD, who began treatment with a bDMARD or tofacitinib after 1 January 2014, and who have been on the medication for more than 6 months. Patients treated with more than one bDMARD before the index event or who had previous diagnoses of Crohn’s disease (K50), ulcerative colitis (K51), ankylosing spondylitis (M45), psoriasis (L40), or psoriatic arthritis (L40.5; M07.0-M07.3) were excluded. Those who did not have continuous management with bDMARD or tofacitinib during the last 6 months of follow-up were also excluded.

The index date was defined as the time of dispensing the first dose of bDMARD or tofacitinib to each patient. From that time on, the patient was followed up for 12 months to identify the resources used during that period and the possible barriers to access to the medication that he/she would have registered.

The following groups of variables were considered:

-Sociodemographic: age, sex, city.
-Clinical: clinical diagnosis (ICD-10 codes), time since diagnosis of the disease (in years), severity of disease at baseline according to Disease Activity Score (DAS28), comorbidities (metabolic, cardiovascular, hepatic, neoplastic, rheumatologic, respiratory, and gastrointestinal), and adverse drug reactions.
-Pharmacological: previous treatment with cDMARDs, bDMARDs, or DMARDs with a specific target, route and frequency of administration, suspension of any of the medications, and the cause of suspension.
-Use of resources: proportion of days covered, cost of treatment, use of other medications related to RA and their cost, number of hospital admissions caused by adverse events, type of adverse event that led to hospitalization, number of hospital admissions due to complications of the disease, type of complication, number of visits for administration of bDMARD, cost per administration (rheumatology care center- IPS-E), number of visits with rheumatology and its cost, number of visits to other medical specialties and their cost, and number of visits to emergency departments. There was no information on the costs of hospitalization or emergency room visits (general hospital).
-Access barriers: time from prescription to drug dispensing and number of days of delay in drug delivery.
-Outcomes: persistence of medication use (the continuation of medication from the index date until 12 months of follow-up with drug claim gaps no greater than 60 days (19)); proportion of days covered, calculated from the days that the patient would have the medication according to the units delivered over the number of days prescribed [21].

The economic variables were identified from the review of patients’ clinical records; information was extracted following the macro-costing method, where only direct costs of the use of services, including those used by the patient (medical consultations, drug application, pharmaceutical expenses, etc.), were considered. The Colombian peso was used with conversion to US dollars under the respective exchange rate according to the Banco de la Republica (Bank of the Republic) (1 USD = 2,920 pesos (December of 2017)). The sources of information were the drug claims database of the logistics operator that dispenses them, the clinical records of IPS-E, and its billing database. Information on the drug claims base and clinical records were validated by the authors.

The statistical package SPSS Statistics version 25.0 (IBM, USA) for Windows was used to analyze the data. Information was gathered by medical professionals following a data extraction template developed in Microsoft Excel. Univariate analyses were run with frequencies and proportions for categorical variables and measures of central tendency, position, and dispersion for the quantitative variables according to their normality (Kolmogorov-Smirnov test).

The study was endorsed by the Bioethics Committee of the Universidad Tecnológica de Pereira in the “risk-free research” category. The principles of confidentiality of information established by the Declaration of Helsinki were respected, and individual informed consent was not required for each patient. In no case were personal data of the research subjects taken.

## Results

A total of 588 patients undergoing bDMARD or tsDMARD treatment for the control of RA were evaluated, with a predominance of females (*n* = 518, 88.1%) and a mean age of 57.3 ± 12.5 years. They were residents of 60 different cities in Colombia. The most recorded diagnosis was seropositive RA (*n* = 441, 75.0%). Regarding the level of activity of RA, by the onset of follow-up, the mean DAS28 score was at 4.8 ± 1.2 points, while at the end the mean was 3.1 ± 1.5 points (*p* < 0.001). Table 1 shows sociodemographic characteristics and data on complications, duration of the disease, and severity according to type of previous therapy. The most frequent cDMARDs previously used were methotrexate (*n* = 542; 92.2%), leflunomide (*n* = 456; 77.6%), chloroquine (*n* = 316; 53.7%), and sulfasalazine (*n* = 300; 51.0%), and the most frequent bDMARDs previously used by patients were etanercept (*n* = 51; 27.1%), adalimumab (*n* = 45; 23.9%), abatacept (*n* = 22; 11.7%), tocilizumab (*n* = 21; 11.2%), rituximab (*n* = 18; 9.6%), certolizumab (*n* = 15; 8.0%), infliximab (*n* = 11; 5.9%), and golimumab (*n* = 5; 2.7%).

**Table 1:** Sociodemographic and clinical characteristics in patients with rheumatoid arthritis, according to the previous use of conventional and biological disease-modifying antirheumatic used prior index date in a cohort of 588 patients diagnosed with rheumatoid arthritis in Colombia, 2014–2018

Variable	Total cohort (*n* = 588) - *n* (%)	Patients with previously bDMARD use (*n* = 188) -*n* (%)	Patients with previously cDMARD use (*m* = 400) - *n* (%)
Female	518 (88.1)	162 (86.1)	356 (89.0)
Age; mean (SD)	57.3 (12.5)	57.5 (11.3)	57.2 (13.1)
Rheumatoid arthritis diagnosis			
Seropositive RA	441 (75.0)	138 (73.4)	303 (75.7)
Unspecified RA	95 (16.2)	35 (18.6)	60 (15.0)
Seronegative RA	52 (8.8)	15 (8.0)	37 (9.3)
Rheumatoid arthritis—years since diagnosis; median (IQR)	9.0 (4–17.2)	12 (7–19)	7 (3–16)
Manifestations associated with rheumatoid arthritis	293 (49.8)	106 (56.4)	187 (46.8)
Sjogren's syndrome	138 (23.5)	56 (29.8)	82 (20.5)
Erosions and joint damage	110 (18.7)	38 (20.2)	72 (18.0)
Ophthalmopathy^a^	76 (12.9)	20 (10.6)	56 (14.0)
Pulmonary involvement^b^	19 (3.2)	8 (0.04)	11 (0.02)
Disease activity (beginning of follow-up) DAS28—mean (SD)	4.8 (1.2)	4.7 (1.2)	4.9 (1.3)

*cDMARDs* conventional disease-modifying antirheumatic drugs, *bDMARDs* biological disease-modifying antirheumatic drugs. *SD* standard deviation. *IQR* interquartile range. ^a^Episcleritis, scleritis, ulcerative keratitis, and uveitis. ^b^Pleurisy interstitial fibrosis, pulmonary nodules

At the beginning of the follow-up, it was found that 32.0% (*n* = 188) of the patients had previously received a first bDMARD therapy, especially etanercept, while all of them had used cDMARD therapy, such as methotrexate and leflunomide. Patients received a mean of 2.0 ± 0.6 medications, and 86 (14.6%) were treated with one medication—tofacitinib *n* = 28 (33.7% of tofacitinib users) and bDMARDs *n* = 58 (11.5% of bDMARDs users), while 384 (65.3%) received two medications, 110 (18.7%) received three medications, and 8 (1, 4%) received four. Table 2 shows the treatment used, with their mean dose, frequency, and route of administration. In addition, 401 (68.2%) patients were receiving acetaminophen simultaneously, 126 (21.4%) received opioids, and 103 (17.5%) received nonsteroidal anti-inflammatory drugs.

**Table 2:** Treatment patterns of a cohort of patients diagnosed with rheumatoid arthritis in Colombia, 2014–2018

Current therapy	*n*	%	Mean dose (mg/day)	Most frequent interval	Most frequent administration route
cDMARDs	502	85.4			
Methotrexate	275	46.8	14.1 ^a^	Weekly	Oral
Leflunomide	240	40.8	20.0	Daily	Oral
Sulfasalazine	49	8.3	1265.3	Every 12 h	Oral
Chloroquine	35	6.0	145.1 ^b^	Daily	Oral
Hydroxychloroquine	18	3.1	277.7	Daily	Oral
Azathioprine	11	1.9	79.5	Daily	Oral
tsDMARD					
Tofacitinib	83	14.1	10.0	Every 12 h	Oral
bDMARDs	505	85.9	mg/application		
Etanercept	100	17.0	50.0	Weekly	Subcutaneous
Rituximab	89	15.1	1752.8	Total cycle	Intravenous
Adalimumab	78	13.3	40.0	Each 15 days	Subcutaneous
Certolizumab	68	11.6	400	Monthly	Subcutaneous
Tocilizumab	59	10	540.7	Monthly	Intravenous
Golimumab	58	9.9	50.0	Monthly	Subcutaneous
Abatacept	52	8.8	596.1	Monthly	Intravenous
Infliximab	1	0.2	200.0	Cycle and every 8 weeks	Intravenous

^a^Average according to dosage interval different from daily. ^b^Dose according to active ingredient. *cDMARDs* conventional disease-modifying antirheumatic drugs, *tsDMARDs* target-specific disease-modifying antirheumatic drugs, *bDMARDs* biological disease-modifying antirheumatic drugs

The mean length of bDMARD or tofacitinib use during the follow-up year was 9.8 ± 1.9 months (with 292.7 ± 61.3 days covered), although with gaps in continuity, because only 12.1% (*n* = 71) received a full 12 months of therapy. A total of 394 (67.0%) patients stopped treatment during this period, while 41 (7.0%) had a change in management and in 83 (14.1%) patients, the bDMARD or tofacitinib, the suspension was permanent. Table 3 shows the causes of the suspension. Table 4 shows the frequency of suspension of each biological drug and the type of change that was undertaken. The mean delay time between the prescription (bDMARDs or tofacitinib) and the first delivery or application was 31.3 ± 23.9 days; only 267 (45.4%) patients had this information reported in the clinical records; for tofacitinib, the mean claim delay was 18.1 ± 9.8 days and bDMARD claim delay 33.2 ± 24.8 days.

**Table 3:** Main causes of discontinuation of therapy with biological and target-specific disease-modifying antirheumatic drugs in a cohort of patients diagnosed with rheumatoid arthritis treated in Colombia, 2014–2018

Discontinuation of therapy	*n* = 395	%
No reason recorded in the medical record	290	66.5
Lack of authorization by health insurer	39	8.9
Adverse events	26	6.0
Lack of therapeutic response	15	3.4
Change of treating physician	3	0.7
Poor patient adherence	1	0.2
Other motives	21	4.8
Medication error	4	0.9
Surgery	4	0.9
Difficulties in assistance for application or drug claim	4	0.9
Death during follow-up	4	0.9
Acute unrelated infections (diarrhea, animal bite, virus)	3	0.7
Gestation	1	0.2
Cancer	1	0.2

**Table 4:** Mean time of use, frequency of suspension or change of each biological and target-specific disease-modifying antirheumatic drug, and replacement medication in a cohort of patients diagnosed with rheumatoid arthritis treated in Colombia, 2014–2018

Initial bDMARD or tsDMARD	Average use (months covered). Mean ± SD	Suspension, change or failure in persistence. [*n* (%)]	Change to (*n*):								
			Etanercept	Rituximab	Tofacitinib	Adalimumab	Certolizumab	Tocilizumab	Golimumab	Abatacept	Infliximab
Etanercept (*n* = 100)	9.36 ± 1.75	88 (88.0)	NA	0	1	0	1	1	0	1	0
Rituximab (*n* = 89)	11.8 ± 1.08	6 (6.7)	0	NA	2	0	1	2	0	1	0
Tofacitinib (*n* = 83)	9.18 ± 2.07	71 (85.5)	1	1	NA	0	0	2	1	0	0
Adalimumab (*n* = 78)	9.58 ± 1.85	67 (85.9)	0	2	1	NA	1	3	0	1	0
Certolizumab (*n* = 68)	9.37 ± 1.83	59 (86.7)	0	1	1	0	NA	1	0	0	1
Tocilizumab (*n* = 59)	9.56 ± 1.85	51 (86.4)	0	0	1	1	3	NA	0	0	0
Golimumab (*n* = 58)	9.83 ± 1.86	46 (79.3)	0	1	0	0	0	2	NA	2	0
Abatacept (*n* = 52)	9.02 ± 1.91	47 (90.3)	1	1	1	0	0	1	0	NA	0
Infliximab (*n* = 1)	9.50 ± NA	1 (100.0)	0	0	0	0	0	0	0	0	NA

*tsDMARDs* target-specific disease-modifying antirheumatic drugs, *bDMARDs* biological disease-modifying antirheumatic drugs, *SD* standard deviation, *NA* not applicable

A total of 35 (6.0%) patients were reported to have had an adverse reaction associated with the use of a bDMARD or tofacitinib, the most frequent being those of infectious origin (*n* = 13; 2.2%), followed by local or injection site-related reactions (*n* = 8; 1.4%), hepatic or gastrointestinal (*n* = 6; 1.0%), cardiovascular (*n* = 3; 0.5%), central nervous system (I = 2; 0.3%), respiratory (*n* = 1; 0.2%), and others, such as dyslipidemia and angioedema in one patient each (0.2%).

The most common comorbidity was type 2 diabetes mellitus in 72 patients (12.2%), many of them had target organ damage as a result of diabetes (*n* = 23; 3.9%); followed by liver disease (*n* = 61; 10.4%), history of a localized solid tumor (*n* = 42; 7.1%), rarely with tumor metastasis (*n* = 2; 0.3%); chronic kidney disease (*n* = 33; 5.6%); chronic obstructive pulmonary disease or asthma (*n* = 49; 8.3%); peripheral vascular disease (*n* = 46; 7.8%); depression or anxiety (*n* = 39, 6.6%); coronary artery disease (*n* = 20; 3.4%); heart failure (*n* = 18; 3.1%); acid-peptic disease (*n* = 11; 1.9%); stroke (*n* = 10; 1.7%); and hematological neoplasias such as lymphomas or leukemia (*n* = 3; 0.5%). The Charlson comorbidity index had a mean of 3.1 ± 1.7 points (range: 1–12 points).

During the follow-up year, on average, patients had 2.8 ± 1.1 consultations with rheumatologists (range: 1–6 visits). A mean of 1.5 visits to other medical specialties was found, the most frequent being to internal medicine, dermatology, and orthopedics. During follow-up, only one patient had a hospitalization related to articular complications of RA while three patients were hospitalized due to adverse events from rituximab (angioedema), golimumab (dyspnea), and tocilizumab (infectious complication), respectively.

Regarding costs, an annual mean of total cost per RA patient of USD 8996.9 ± 2172 was found, and the total expense of the 588 patients was USD 5,257,256. Of the total cost, 97.2% was associated with pharmaceutical expenditure, and of this, 92.1% was spent on bDMARDs or tofacitinib, while the remaining cost was associated with cDMARDs, analgesics, and other medications. Only 1.7% of expenses was associated with the application of subcutaneous medications or infusions, and 1.1% represented the cost of medical visits (see Table 5).

**Table 5:** Direct costs associated with pharmacological treatment and medical care of a cohort of patients diagnosed with rheumatoid arthritis treated with biological and target-specific disease-modifying antirheumatic drugs in Colombia

Direct costs	Proportion of expenditure (%)	Mean costs (USD)/patient/year	Total costs (USD)
Total costs—direct care and treatment expenses			
*Total annual expenses (n = 588)*	100.0	8997	5,257,256
*Total medication expenses*	97.2	8744	5,141,536
*Annual cost of cDMARDs (n = 588)*	2.0	183	107,370
*Annual cost of bDMARDs or tofacitinib (n = 588)*	92.1	8289	4,874,055
Annual cost of bDMARDs (*n* = 505)		8604	4,344,786
Annual cost of tofacitinib (*n* = 83)		6,377	529,269
*Annual cost of other medications (analgesics) (n = 588)*	0.4	36	20,907
*Cost of bDMARDs or tofacitinib, initiated after a change in therapy (n = 41)*	2.6	3395	139,203
*Total expenditure on subcutaneous medication application or infusions*	1.7	157	92,370
*Total expenditure on medical visits and care*	1.1	96	56,228

*USD* United States dollar, *DMARDs* disease-modifying antirheumatic drugs, *cDMARDs* conventional disease-modifying antirheumatic drugs, *bDMARDs* biological disease-modifying antirheumatic drugs

## Discussion

RA is a disease with a high burden of comorbidities, complications, and worse health-related quality of life and is associated with elevated healthcare costs. The bDMARDS or tofacitinib medications are indicated for those with significant progression of the disease and when there is a need for alternatives to achieve low levels of activity and remission [22]. However, these medications are related with a significant increase in direct costs and with all medications there is a risk of some adverse reactions [16, 23]. The present study describes the clinical characteristics, treatment patterns, persistence, and direct healthcare costs associated with bDMARD or tofacitinib treatment in RA patients in Colombia in a real-life context.

The patients evaluated in this cohort had a high frequency of other diseases as measured with the Charlson comorbidity index, which directly impacts the probability of death when compared with cases whose score is 0 [24]. Diabetes mellitus was diagnosed in 12% of our patients, and peripheral vascular disease, coronary disease, and heart failure were common, which have been widely recognized as the main risk factors related to premature mortality in RA patients [25, 26]. This mortality is explained mainly by accelerated atherosclerosis and by its suboptimal medical management in different cohorts, which not only impacts health-related quality of life and survival but also raises direct medical expenditures unrelated to RA [27–29].

The patients included had a mean history of 10 years with RA, and almost half of them already had associated complications, which can be explained by the inclusion criteria, leading to a population who already had greater activity of the pathology. This made them candidates to receive bDMARDs or tofacitinib, in accordance with their high DAS28 score [5, 30]. The start of the observation period of each subject was the time of first prescription of these drugs, and thus the level of activity and complications should be correlated with the therapy they had been receiving previously. It is relevant that 32% of patients had already received a bDMARD and that nearly 85% continued receiving a cDMARD concomitantly. The reduction in the DAS28 score during the observation period is a sign of the effectiveness of the treatment [5, 31].

In view of the above, the mean time of use of the drugs was approximately 10 of the 12 months of follow-up (83.3% of the time covered by the drug).This gap can be explained by the proportion of patients who discontinued the drug with or without a recorded reason and the difficulties accessing medications associated with authorizations by the insurer, which affects the continuity of long-term therapy according to the requirements of the patient and may impact the outcomes and activity of the disease [32, 33].

The mean annual direct cost of treatment with bDMARDs or tofacitinib for each patient was close to USD 9000, a very high value compared with the mean necessary to cover the health costs of a Colombian, which according to the Ministry of Health of Colombia was 277 USD/year in 2017 [34]. However, it is lower than that found by the systematic review by Hresko et al. in the United States, where they estimated a direct cost at USD 36,053 for each RA patient treated with bDMARDs [16], probably explained by the local prices of medications, medical consultations, and procedures required. The same study estimated that starting therapy with a bDMARD increases the annual direct cost for pathology control by approximately 444% [16]. In the study published by Leon et al. in Spain, it was estimated that a patient with RA treated without a bDMARD had a direct cost of approximately 5% of the total annual cost, while receiving a bDMARD raised this value to more than 90% of the annual total, a phenomenon similar to that found in this study [35].

The cost of tofacitinib compared with bDMARDs was lower in the evaluated population, mainly explained by a lower monthly value paid for the medicine; however, this phenomenon might differ according to the environment studied. For example, similar results were observed by Chastek et al., who reported that the lowest cost of treatment in rheumatoid arthritis included tofacitinib, infliximab, and abatacept, according to the information of more than 20,000 clinical records from United States [36]. Conversely, a cost-minimization analysis conducted in Brazil, which reviewed national databases that provide information of healthcare resources, reported unlike results being tocilizumab the lowest treatment under the scenery of population under 60 kg of weight [37].

The relationship between the high costs of pharmacological treatment of RA patients and the lack of persistence or adherence throughout follow-up demonstrates the difficulties that health systems have in covering this pathology, especially in developing countries, which have limited resources and which need to select cost-effective therapies based on their own analysis that guarantee access and coverage that are also safe and improve the quality of life of those who require them [38, 39]. Undoubtedly, the problems of lack of persistence in the use of antirheumatic drugs deserve special attention, which leads to the need to search for strategies that identify the causes and improve adherence to treatment. In addition, the management of comorbidities and complications that, according to some studies, may be responsible for up to 44% of the total direct costs of patients with RA should be considered [40]; this condition was not measured in this study. Another point of interest was the indirect costs, which could not be identified and quantified in this study and that in others has been estimated to represent approximately 25% of the total costs of the disease, hence the importance of choosing effective treatments that achieve their therapeutic objective [41, 42].

Adverse reactions were relatively infrequent in this cohort of patients, despite strict follow-up through an active pharmacovigilance program, which can be explained by the efforts of the care staff in achieving a high adherence to the administration protocols of each drug and by the specific selection of the drug adjusted to the individual needs [14]. However, the short follow-up time did not allow the identification of reactions that may be associated with long-term use.

The limitations of this study include the fact that its conclusions are applicable only to patients with similar insurance and health service provision characteristics in Colombia. In addition, the actual frequency of hospitalizations and emergency department care for all patients is unknown, as are the direct costs associated with care during hospital stays, and laboratory and imaging test costs were not included, situations that add up to the direct costs of the disease. Nor were indirect costs related to loss of labor productivity or disabilities obtained, nor those associated with the management of other pathologies or complications derived from RA. The follow-up was relatively short, so the conclusions about the longer term should be made with caution. Finally, it was not possible to find out the reasons for lack of continuity or suspension of therapy in all subjects. The study’s strengths include the complete clinical follow-up of a cohort of RA patients undergoing treatment with bDMARDs or tofacitinib, as well as the estimation of the direct costs not only of pharmacological therapy but also of the administration of drugs and medical visits.

It can be concluded that patients with RA treated with bDMARDs or tofacitinib represent a significant economic burden to the health system, especially in the costs derived from pharmacological treatment, in addition to the high frequency of comorbidities. On the other hand, relatively few problems of adherence can be identified, since most patients received the therapy month after month throughout the year. However, this should encourage the development of strategies to improve adherence in those who do not comply, since nonadherence can slow the remission of the disease and promote the appearance of complications, which should be quantified and costed. This study shows the direct costs of treatment incurred by insurers, data that may be useful for decision-making that favors outcomes, ensuring the effectiveness, safety, and proper use of economic resources for AR care.

## Data Availability

Protocolos.io
